# Infantile Abdominal and Pelvic Lipoblastomas: A Case Series

**DOI:** 10.1055/s-0039-1694060

**Published:** 2019-11-28

**Authors:** Riccardo Guanà, Salvatore Garofalo, Luisa Ferrero, Maria Grazia Cortese, Luca Lonati, Elisabetta Teruzzi, Eleonora Basso, Isabella Morra, Riccardo Lemini, Nicola Sardi, Fabrizio Gennari

**Affiliations:** 1 Division of Pediatric Surgery, Regina Margherita Children's Hospital, Turin, Italy; 2 Department of Pediatric Surgery, Ospedale Infantile Regina Margherita, Turin, Piemonte, Italy; 3 Department of Pediatric Surgery, Regina Margherita Children's Hospital, Piazza Polonia, Turin, Italy; 4 Department of Paediatric Haematology-Oncology, Ospedale Infantile Regina Margherita, Turin, Piemonte, Italy; 5 Department of Anatomo-Pathology, Ospedale Infantile Regina Margherita, Turin, Piemonte, Italy; 6 Surgery, Section of Colon and Rectal Surgery, Mayo Clinic Hospital Jacksonville, Jacksonville, Florida, United States; 7 Department of Paediatrics, Ospedale SS Annunziata di Savigliano, Savigliano, Piemonte, Italy

**Keywords:** lipoblastoma, surgery, children

## Abstract

Lipoblastomas are rare benign mesenchymal tumors that arise from embryonal fat cells.

They are usually discovered in infants and children under 3 years of age, and mostly occur in the trunk (from 10 to 60%, depending on the study) and extremities (from 40 to 45%), while head and neck localizations are rare, with only five cases described to date.

We report on three cases of lipoblastomas in infants younger than 4 years, with unusual localizations: one intra-abdominal, discovered during a laparotomy for an intussusception; one pelvic, misdiagnosed as an ovarian mass; and one gluteal with a pelvic extension.

All children underwent magnetic resonance imaging as preoperative workup. All tumors were completely resected with free surgical margins and ultrasonographic follow-up was uneventful for all patients.

## Introduction

Lipoblastomas are rare benign mesenchymal tumors that arise from embryonal fat cells.

They are usually discovered in infants and children under 3 years of age, and mostly occur in the trunk (from 10 to 60%) and extremities (from 40 to 45%), while head and neck localizations are rare, with only five cases described to date. 1

They are classified as benign adipocytic tumors, along with lipomas, lipomatosis, lipoblastomatosis, angiolipomas, and myolipomas.

Intermediate (locally aggressive) adipocytic tumors are atypical lipomatous tumors and well-differentiated liposarcoma, while malignant tumors are dedifferentiated liposarcoma, myxoid liposarcoma, pleomorphic liposarcoma, and mixed-type liposarcoma.

So far, 85 cases of lipoblastoma have been described in childhood, of which 12 presented with an abdominal mass. 1

The treatment of choice is complete surgical excision.

Only five cases of liposarcoma have been reported in childhood, of which two had arisen from the porta hepatis.

In this report, we describe three cases of lipoblastoma in infants below 4 years of age.

## Case Presentation

### Case 1

**Fig. 1:**
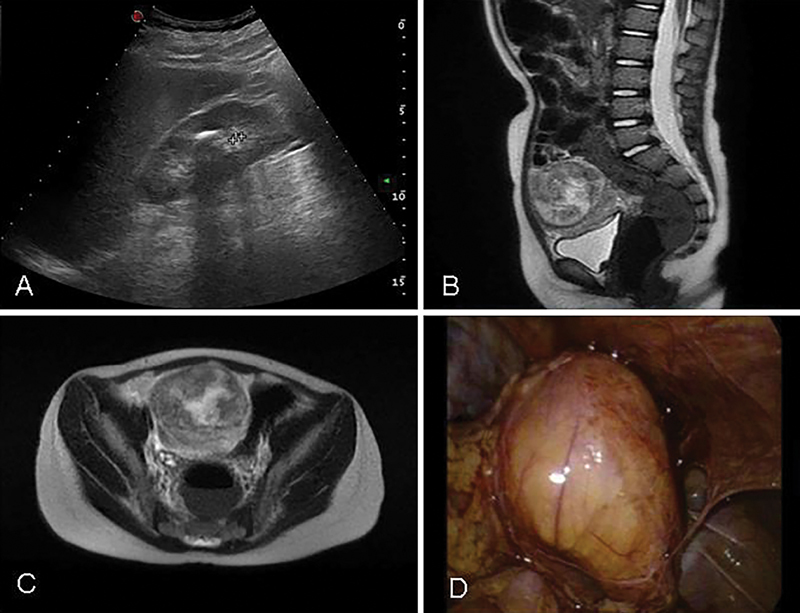
Abdominal ultrasound showing a 6 cm right-sided ovarian mass that was consistent with teratoma ( **A** ). To better characterize the mass, an abdominal magnetic resonance imaging was performed and the initial diagnosis was confirmed ( **B, C** ). Laparoscopic view of the mass ( **D** ).

A 4-year-old female was hospitalized for a suspected ovarian mass. The child complained of recurrent abdominal pain and dysuria for 1 month; an abdominal ultrasound and magnetic resonance imaging (MRI) showed a 6 cm right-sided ovarian mass that was suspected to be a teratoma ( Fig. 1A–C ). The girl underwent laparoscopy with 5 mm instruments. However, no ovarian mass was found, but an 8-cm exophytic lesion was discovered, arising from the retroperitoneum, on the right side of the pelvis, adjacent to the bladder with multiple adhesions with the ascending colon and the uterus ( Fig. 1D ). The tumor was completely resected by laparoscopy with no residual lesions, and the postoperative course was uneventful.

**Fig. 2:**
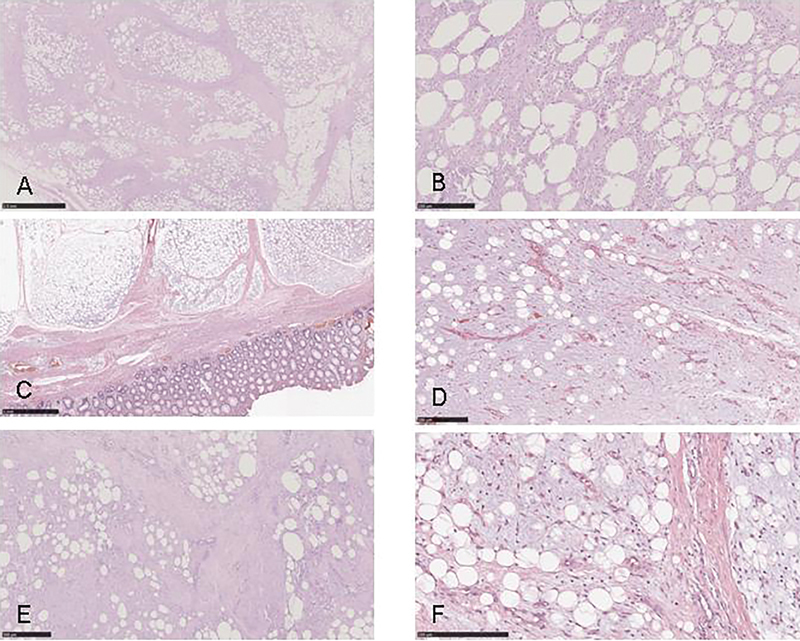
Lipoblastoma histology: multilobulated soft tissue lesion comprising groups of peripherally lipoblast that are separated by fibrous septa and a distinct peripheral pseudocapsule was typical of all cases. Hemosiderin staining and chronic inflammation with extensive liponecrosis were present in patient 1( **A** , **B** ) and signs of vascular congestion were present in patient 2 ( **C** , **D** ). Hemosiderin staining and chronic inflammation were particularly represented in patient 3 ( **E** , **F** ).

Pathological examination revealed a solid mass composed of small lobules of mature and immature fat cells separated by fibrous septa, and containing small and dilated blood vessels (lipoblastoma) ( Fig. 2A, B ).

### Case 2

A 2-year-old otherwise healthy female presented with acute abdominal pain and reddish jelly-like stool.

**Fig. 3:**
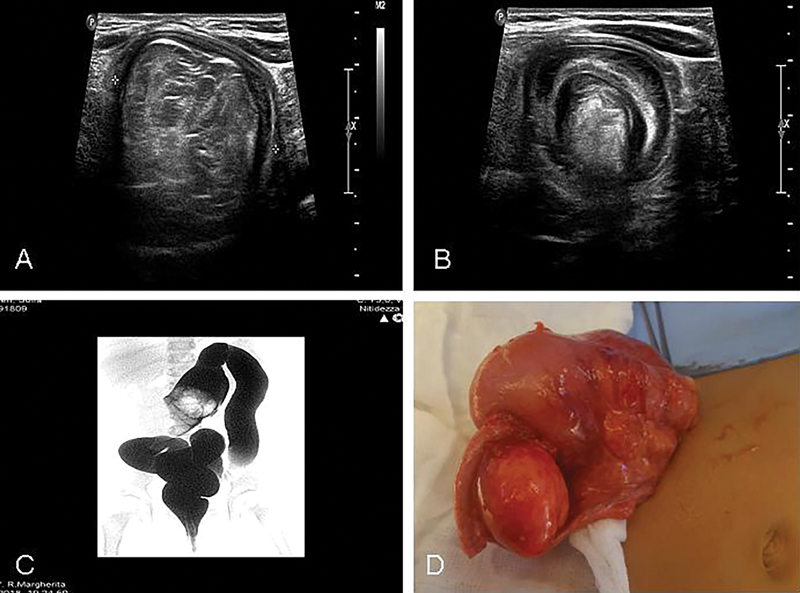
Abdominal ultrasound confirming the intussusception ( **A, B** ). Water-soluble contrast enema was performed but the result was ineffective ( **C** ); hence, the patient underwent a surgical exploration ( **D** ).

On abdominal ultrasound, an ileocecal intussusception was confirmed ( Fig. 3A, B ). Water-soluble contrast enema was performed but was ineffective ( Fig. 3C ); hence, the patient underwent surgical exploration via transverse right-sided supraumbilical laparotomy ( Fig. 3D ). An ileo-appendico-ceco-colic intussusception was found, along with a 4 cm mass as a lead point, located at 1 cm to the ileocecal valve. The mass was firm, round, yellow, similar to a lipoma, with adhesions to the ilocecal angle. Enlargement of mesenteric lymph nodes was also found. Due to the impossibility of manually reducing the intussusception, we opted for an en bloc ileocecal resection including the above-mentioned mass. Finally, lymph node sampling and an end-to-end ileocolic anastomosis were performed.

The histology report described a lipoblastoma with signs of vascular congestion and infiltration of the bowel wall ( Fig. 2C, D ); the removed lymph node was found to be normal.

### Case 3

**Fig. 4:**
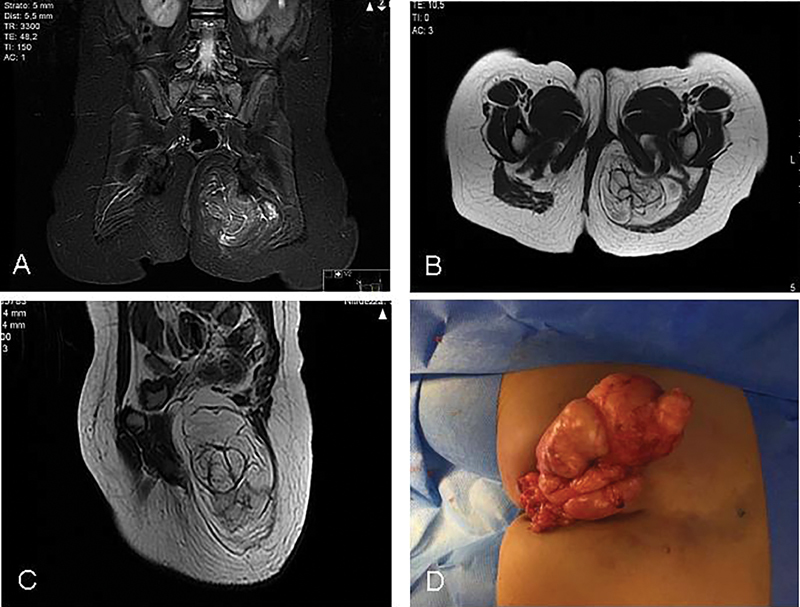
Magnetic resonance imaging showed a multilobulated epifascial lesion measuring 8 × 6 cm in the context of the gluteal muscle that deepened in the pelvis ( **A** – **C** ). Complete surgical excision was achieved via a posterior–sagittal approach ( **D** ).

A 2-year-old female presented with a gluteal mass with slow and continuous growth. MRI showed a multilobulated epifascial lesion measuring 8 × 6 cm, in the context of the gluteal muscle extending to the pelvis ( Fig. 4A–C ). The child underwent complete surgical excision of the tumor including the overlying skin. Intraoperatively, an encapsulated multilobulated subcutaneous fatty tumor was discovered ( Fig. 4D ). Histology revealed a lipoblastoma with chronic inflammation ( Fig. 2E, F ).

In all cases, immunohistochemistry showed primitive mesenchymal cells reactive for Desmin, adipocytes, and vascular endothelial cells positive for CD34, and adipocytes also positive for S100 protein. A follow-up at 12 (Case 3), 14 (Case 2), and 16 (Case 1) months showed all three children doing well. There is no evidence of recurrence, either clinically or on ultrasound.

## Discussion

Lipoblastomas are soft tissue tumors composed of embryonal/fetal fat and characterized by a benign nature, early presentation, male predominance, and rapid growth. 2

It mostly occurs in infants and children under 3 years of age, and the most common locations are extremities and trunk, followed by head and neck. 3

The age at presentation ranges from 1 month to 14 years (median: 2 years).

Sometimes lipoblastoma arises as an abdominal mass from the retroperitoneum, as in our first case, involving the mesentery or the omentum, or acutely as a lead point of an intussusception, as in our second case.

In 1926, Jeff et al 1 first described lipoblastoma as an atypical *lipoma* that consists of cells resembling embryonic fat (lipoblasts).

Two variants are described: lipoblastoma (a well *circumcised* and capsulated tumor) and lipoblastomatosis (a noncapsulated multicentric and infiltrative tumor). Both are rare and benign *mesenchymal tumors* .

Lipoblastomatosis has the same demographic characteristics as lipoblastoma but is more common in the head-and-neck area.

Myxoid liposarcoma is the most common liposarcoma subtype, usually occurring in extremities (thigh), mostly in adolescent males, at a median age of 16 years.

It has an excellent prognosis after surgery, differing from the pleomorphic subtype, more aggressive and with poor prognosis.

Its cytological findings are adipocytes, lipoblasts, and spindle cells that show moderate cytological atypia immersed in a vascularized myxoid stroma. 5

Of the five cases of liposarcoma reported in childhood, three children died, one of them due to a recurrence 12 years after an initial response to surgical excision combined with irradiation and chemotherapy. 2

In all studies reported in the literature, MRI is the diagnostic tool of choice for the evaluation of tumor extension and to distinguish lipoblastoma from normal subcutaneous fat.

Lipoblastomas appear in fact as lobulated lesions characterized by high signal intensities.

Tumor size may vary from few centimeters to 20 to 25 cm.

Treatment of choice is complete surgical resection with preservation of vital organs.

All tumors described so far, presented with lobulated appearance, were soft in consistency and had a yellowish surface. The histology consists of multilobulated soft tissue lesion comprising groups of peripherally arranged immature lipoblast that is separated by thin fibrous septa and a distinct peripheral pseudocapsule.

Diagnosis is typically made after pathological examination of the operative specimen, as in our cases.

Immunohistochemistry (CD34, S100 protein, and Desmin) is usually performed in all studies. Primitive mesenchymal cells are reactive for Desmin, adipocytes and vascular endothelial cells are positive for CD34, and adipocytes are also positive for S100 protein.

The recent use of fine-needle aspiration cytology has proven to be helpful for the diagnosis of lipoblastomas, as mutations involving chromosome 8 (8q11–13 rearrangement involving the PLAG1 gene) have been found in 70% of patients. 5

This examination could be helpful in primary or recurrent tumors with diagnostic dilemmas.

The long-term prognosis for lipoblastoma is good; metastases have never been reported and the potential recurrence (ranging from 9 to 22%, depending on the study) is due to incomplete initial excision of the tumor. 4 For example, in the study of Abdul-Ghafar, 6 a 14-year-old girl with a mediastinal lipoblastoma with infiltrative margins developed four separate recurrences and underwent repeated resections.

In lipoblastomatosis the resection is more demanding, due to the multicentric type of lesions and irregular margins, and a higher rate of recurrence is noted. 6

Furukawa et al described a successful laparoscopic removal of a giant omental lipoblastoma in a 4-year-old girl.

They concluded that for large abdominal tumors in children also, the laparoscopic approach could be recommended as the first procedure when the tumor is preoperatively considered to be benign and resectable. 7

Reported surgical complications associated with tumor resection were chronic bilateral venostasis after excision, internal iliac artery, and vein reconstruction at resection, and one patient that developed septic shock died; all tumors were retroperitoneal lipoblastomas. 4

All authors agree that a follow-up of at least 5 years is essential.

Most of the studies did not report any recurrence during the follow-up period.

## Conclusions

Lipoblastoma is an uncommon benign soft tissue tumor, sometimes arising from the retroperitoneum.

Lipoblastoma usually manifests with local swelling but may also be an incidental diagnosis after surgical resection of a mass or intussusception.

Prognosis is generally good and local recurrence is rare, with no risk of metastatic dissemination. However, prolonged ultrasonographic follow-up is recommended.

Radical surgical excision is the current standard of care.

Surgery should be accurate and complete to avoid potential recurrence.

## Notes

New Insights and the Importance for the Pediatric Surgeon Case gallery, tretament option, and literature review of a very rare lesion in children.
